# Supplementary figures and images for: A Plasmodium falciparum MORC protein complex modulates epigenetic control of gene expression through interaction with heterochromatin
Source: eLife. 2024 Oct 16;12:RP92201. doi: 10.7554/eLife.92201 (PMC11483127; doi:10.7554/eLife.92201)

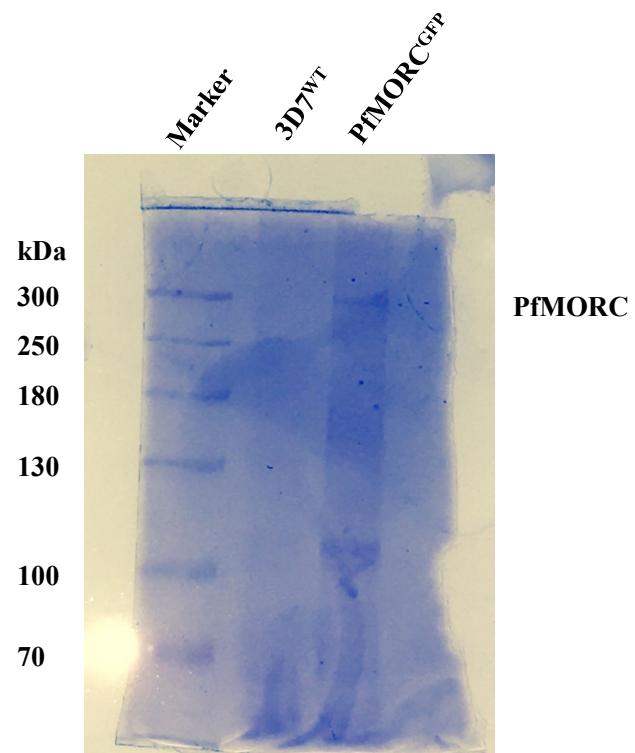

Supplement: Figure 1—figure supplement 1—source data 1. [file elife-92201-fig1-figsupp1-data1.zip › Figure 1-figure supplement 1A-Source Data 1 Uncropped and Labelled SDA-PAGE Gel.pdf]

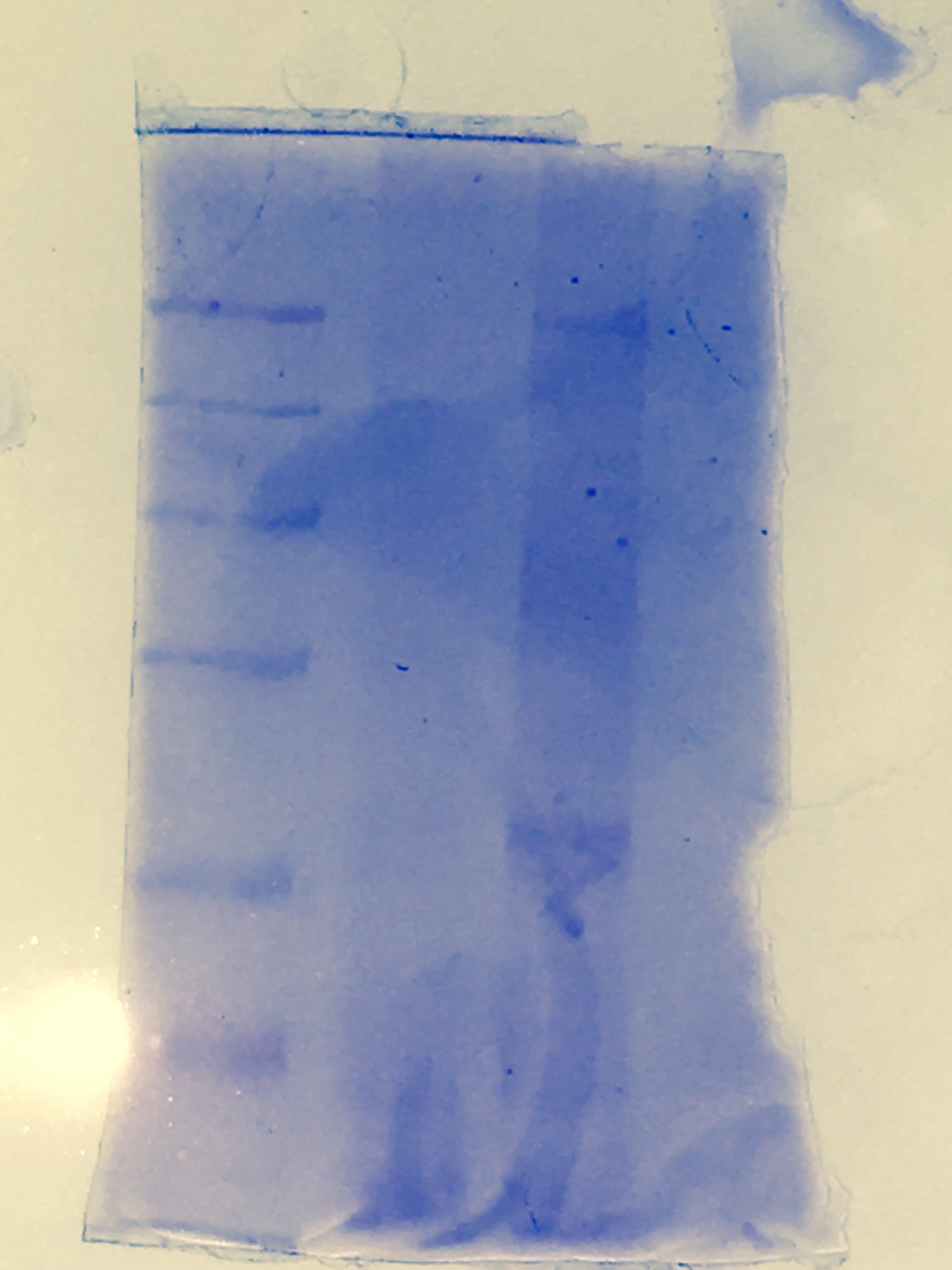

Supplement: Figure 1—figure supplement 1—source data 2. [file elife-92201-fig1-figsupp1-data2.zip › Figure 1-figure supplement 1A-Source Data 2 Raw Unedited SDA-PAGE Gel.JPG]
